# Characterization of and genetic variation for tomato seed thermo-inhibition and thermo-dormancy

**DOI:** 10.1186/s12870-018-1455-6

**Published:** 2018-10-11

**Authors:** Nafiseh Geshnizjani, Farshid Ghaderi-Far, Leo A J Willems, Henk W M Hilhorst, Wilco Ligterink

**Affiliations:** 1 Wageningen Seed Lab, Laboratory of Plant Physiology, Wageningen University, Droevendaalsesteeg 1, NL-6708 PB Wageningen, The Netherlands; 2 Department of Agronomy, Gorgan University of Agricultural Sciences and Natural Resources, Gorgan, Iran

**Keywords:** *Solanum lycopersicum*, *Solanum pimpinellifolium*, Thermo-inhibition, Thermo-dormancy, QTL analysis

## Abstract

**Background:**

Exposing imbibed seeds to high temperatures may lead to either thermo-inhibition of germination or thermo-dormancy responses. In thermo-inhibition, seed germination is inhibited but quickly resumed when temperatures are lowered. Upon prolonged exposure to elevated temperatures, thermo-dormancy may be induced and seeds are not able to germinate even at optimal temperatures. In order to explore underlying physiological and molecular aspects of thermo-induced secondary dormancy, we have investigated the physiological responses of tomato seeds to elevated temperatures and the molecular mechanisms that could explain the performance of tomato seeds at elevated temperature.

**Results:**

In order to investigate how tomato seeds respond to high temperature we used two distinct tomato accessions: *Solanum lycopersicum* (cv. Moneymaker) (MM) and *Solanum pimpinellifolium* accession CGN14498 (PI). MM seeds did not germinate under high temperature conditions while seeds of PI reached a maximum germination of 80%. Despite the high germination percentage of PI, germinated seeds did not produce healthy seedling at 37 °C. By using a candidate gene approach we have tested if similar molecular pathways (abscisic acid (ABA) and gibberellic acid (GA)) present in lettuce and Arabidopsis, are regulating thermo-inhibition and thermo-dormancy responses in tomato. We showed that the ABA biosynthesis pathway genes *NCED1* and *NCED9* were upregulated whereas two of the GA-biosynthesis regulators (*GA3ox1* and *GA20ox1*) were downregulated in tomato thermo-dormant seeds at elevated temperature. To identify novel regulators of tomato seed performance under high temperature, we screened a Recombinant Inbred Line (RIL) population derived from a cross between the two tomato accessions MM and PI for thermo-inhibition and dormancy induction. Several QTLs were detected, particularly for thermo-dormancy, which may be caused by new regulators of thermo-inhibition and thermo-dormancy in tomato.

**Conclusions:**

None of the genes studied in this research were co-locating with the detected QTLs. The new QTLs discovered in this study will therefore be useful to further elucidate the molecular mechanisms underlying the responses of tomato seeds to high temperature and eventually lead to identification of the causal genes regulating these responses.

**Electronic supplementary material:**

The online version of this article (10.1186/s12870-018-1455-6) contains supplementary material, which is available to authorized users.

## Background

Seed germination is the start and end of the life cycle of most flowering plants and is, therefore, a critical step in plant development and growth. Although rapid seed germination directly after sowing is a desirable trait for seeds from the producer’s point of view, it can be an undesirable trait at times [1]. Generally, seeds are able to sense unfavourable environmental conditions and may, thus, postpone germination until conditions are more favourable. Such a physiological response is called dormancy [2]. The occurrence of dormancy is heavily influenced by environmental factors. One of the environmental factors which are becoming more relevant in the light of global warming is elevated temperature [3, 4]. Therefore it is of great importance to study the physiological and molecular background of seed germination and dormancy in response to the adverse effect of high temperatures in more detail. Elevated temperatures not only influence post-germination processes, but may also directly affect germination. Exposure of seeds to high temperature may result in certain physiological responses, called ‘thermo-dormancy’ and ‘thermo-inhibition’, in order to prevent seedling damage. Thermo-inhibition refers to the fact that seeds will halt germination at high temperature, but will immediately germinate upon encountering optimal temperatures. Thermo-dormancy is defined as dormancy that is induced by high temperatures [5, 6]. In this case seeds will not germinate at high temperatures, but also not when the seeds are exposed to lower/optimal germination temperatures. This implies that prolonged imbibition at high temperature results in the induction of dormancy.

Many studies have shown that germination of species such as sunflower (*Helianthus L.*), carrot (*Daucus carota*), *Arabidopsis thaliana* and lettuce (*Lactuca sativa*) is inhibited if they are exposed to high temperatures during imbibition [7–10]. Genetic, molecular and physiological analysis have provided insights into the mechanisms of seed germination and dormancy [11]. Thermo-inhibition and thermo-dormancy can be alleviated by environmental factors such as light and nitrate, or by applying GA, inhibitors of ABA biosynthesis, or ethylene [2, 9, 12–15].

Combining genetic and physiological analysis may improve the understanding of the molecular mechanisms underlying seed germination and dormancy [16, 17]. The existing natural variation in plants for seed germination behaviour has been used for genetic analysis and identification of Quantitative Trait Loci (QTLs). Many studies have been reported regarding the genetic aspects of thermo-inhibition and thermo-dormancy in seeds resulting in several QTLs in both model plants such as Arabidopsis and crops such as rice and lettuce [18–20].

Abscisic acid (ABA) and gibberellic acid (GA) are phytohormones that play critical roles in the life cycle of plants, including seed germination and dormancy. ABA has been shown to be a major inhibitor of seed germination and it has an important role in the induction of primary and secondary dormancy in seeds. The ABA biosynthesis pathway is sensitive to several environmental factors and this is one way in which different environments result in different levels of dormancy [21, 22]. Conversely, by application of exogenous GA or by increasing the GA synthesis in seeds, dormancy can be alleviated in many plant species [12, 23]. Thus, germination is regulated by a balance between synthesis and catabolism of ABA and GA. Several genes are known which function in ABA (9-cis-epoxycarotenoid dioxygenase (*NCEDs*) and *ABAs*) and GA (*GA3ox1*, *GA3ox2* and *GA2ox1*) biosynthetic pathways and which may, thus, affect the inhibition of germination caused by high temperatures [24, 25]. In general, when seed germination is inhibited, ABA-associated genes are up-regulated and GA-related genes are suppressed. In Arabidopsis, it has been indicated that high temperature results in the accumulation of ABA in seeds by increased expression of *NCED2*, *NCED5* and *NCED9* and reduced GA content by repression of *GA20ox* and *GA3ox* [9]. In lettuce, it has been shown that expression of the *LsNCED4* gene, which is involved in ABA biosynthesis, was highly induced under high temperature [6].

Further studies have indicated that the maturation, germination and dormancy of seeds may be regulated by an interaction between phytohormones (e.G. *aba*, GA) and a network of transcription factors [26, 27]. For example, *FUSCA3 (FUS3)* is a B3-domain transcription factor which plays a critical role in hormonal biosynthesis and signalling pathways and, consequently, in the life cycle of plants. Under stress conditions such as high temperature, *FUS3* can induce dormancy in seeds by increasing and decreasing ABA and GA biosynthesis, respectively [11, 28, 29]. In Arabidopsis, it was reported that high temperature greatly increased the expression of *FUS3*. Overexpression of *FUS3* results in delayed seed germination at high temperature (32 °C), while mutant lines (*fus3*) are tolerant to that high temperature [30]. Microarray analysis has revealed the direct effect of *FUS3* on the expression *NCED5* and *NCED9* and several GA biosynthesis genes. This implies that thermo-dormancy may be the consequence of an increase in *NCED5* and *NCED9* expression in Arabidopsis [31, 32]. A similar effect of high temperature on the expression of *FUS3* was found in lettuce (*LsFUS3*) and it is possible that also in lettuce FUS3 has a direct effect on the expression of *LsNCED4* [1].

Ethylene has been shown to be one of the promotors of seed germination [14, 33]. It functions in breaking seed coat imposed dormancy in species such as *Rumex crispus* and Arabidopsis [34, 35]. 1-aminocyclopropane-1-carboxylic acid (ACC) synthase (*ACS*) is one of the genes which is involved in ethylene biosynthesis. In lettuce [36], chickpea (*Cicer arietimum*) [37], sunflower [38] and tomato [39] it was reported that either ethylene or its biosynthetic precursor ACC could break thermo-dormancy.

Several reports have presented putative hormonal and molecular mechanisms by which seeds may perceive environmental signals and regulate dormancy and germination [40, 41]. Despite many studies on the mechanisms of thermo-inhibition and thermo-dormancy in crops such as lettuce, little is known about the germination behaviour and regulation of tomato seeds at high temperature. The objective of this study was to investigate the genetic variation of thermo-inhibition between two tomato accessions: *Solanum lycopersicum* (cv. Moneymaker) (MM) and *Solanum pimpinellifolium* (PI). Furthermore, we have used a candidate gene approach to see if similar molecular mechanisms as in lettuce and Arabidopsis are likely to regulate thermo-inhibition and thermo-dormancy in tomato. Finally, a Recombinant Inbred Line (RIL) population derived from a cross between the MM and PI tomato accessions [42] has been used to perform QTL analysis and detect new QTLs and potential new regulators of seed germination and dormancy in tomato under high temperature.

## Results

### Germination of the cultivated tomato accession *Solanum lycopersicum* (cv. Moneymaker) seeds in response to optimal and high temperature

**Fig. 1:**
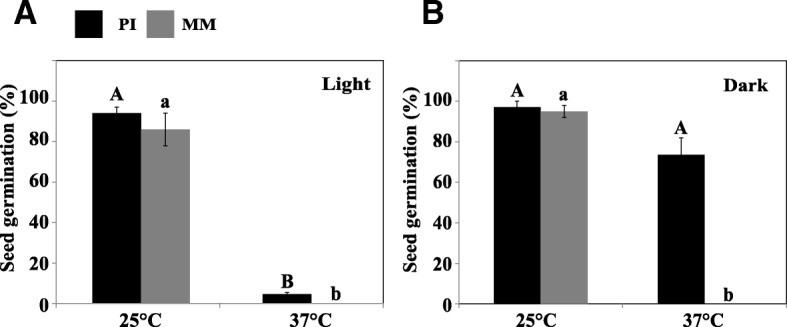
Germination of *Solanum lycopersicum* (cv. Moneymaker) (MM) and *Solanum pimpinellifolium* (PI) seeds at optimal (25 °C) and high temperature (37 °C) in the light (**a**) and in darkness (**b**). Statistical analysis was performed within each genotype

Seeds of MM plants germinated to around 90% at 25 °C in both light and dark conditions. However at high temperature (37 °C) MM seeds neither germinated at light nor at dark (Fig. 1a, b). Thus seeds of the MM genotype were unable to germinate at 37 °C.

**Fig. 2:**
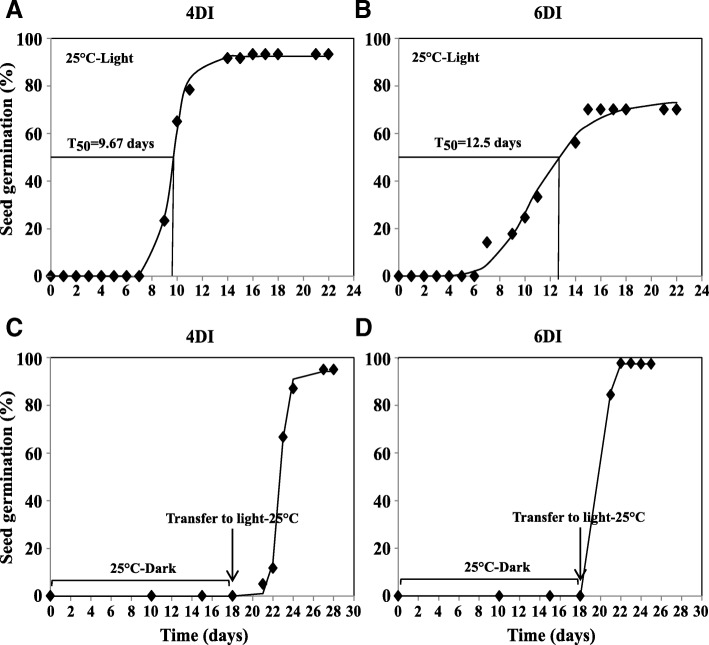
**a** and **b**, Germination percentage of (*Solanum lycopersicum* cv. Moneymaker) 4DI and 6DI seeds at 25 °C with light, respectively; **c** and **d**, germination percentage of the same 4DI and 6DI seeds, respectively, at 25 °C without light and post transferring to light after 18 days of dark imbibition

To investigate if the inhibitory effect of the temperature is either thermo-inhibition or thermo-dormancy, the non-germinated MM seeds were transferred to the optimal germination temperature (25 °C) in the presence and absence of light after 4 days (4DI) and 6 days (6DI) of imbibition at 37 °C. In the presence of light at 25 °C, seeds which had been imbibed at 37 °C for 4 days (4DI), germinated more and faster than seeds which had been imbibed for 6 days at 37 °C (6DI) (Fig. 2a, b). Neither 4DI nor 6DI seeds germinated without light at 25 °C. Nevertheless, when 4DI and 6DI seeds after 18 days of dark imbibition were transferred to light conditions, they started to germinate (Fig. 2c, d). Interestingly, the 6DI seeds which germinated more slowly and to a lower percentage in dark, could immediately germinate after transfer to light and reached 100% of germination after a few days. However, germination of 4DI seeds was delayed by 3 days after transfer to optimal temperature in the light. It took 9 days for these seeds to reach ~ 100% of germination, starting from the transfer date. It may be concluded that at high temperature thermo-dormancy was induced concomitantly with the induction of light sensitivity.

### Endospermic dormancy in MM tomato seeds

**Fig. 3:**
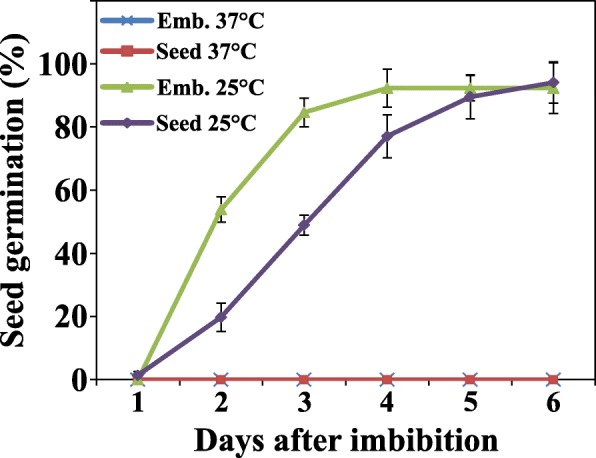
Germination of seeds and separate embryos (Emb.) of tomato seeds (*Solanum lycopersicum* cv. Moneymaker) under normal (25 °C) and high temperature (37 °C)

Stiffness of the endosperm can be one of the major inhibiting factors in germination of tomato seeds [15] and therefore we investigated the role of the endosperm in thermo-dormancy of tomato seeds. Germination of MM embryos which were dissected from the seeds was assessed at 25 °C and 37 °C. The absence of seed coat and endosperm had some effect on germination speed at 25 °C. Embryos showed uniformly and rapid radicle growth in almost 90% of the seeds after three days (Fig. 3). However, removing the endosperm did not promote seed germination at high temperature and seeds became dormant in that condition even without seed coat and endosperm (Fig. 3, Additional file 1: Figure S1). Our results suggest that the thermo-dormancy induced in tomato seeds is not related to the inhibition of endosperm weakening but to embryo dormancy. Thus, contrary to general consensus of tomato seed dormancy being coat-imposed we here propose that tomato seed dormancy also has a component of physiological dormancy located in the embryo.

### Required time to induce thermo-dormancy in tomato seeds (MM)

**Fig. 4:**
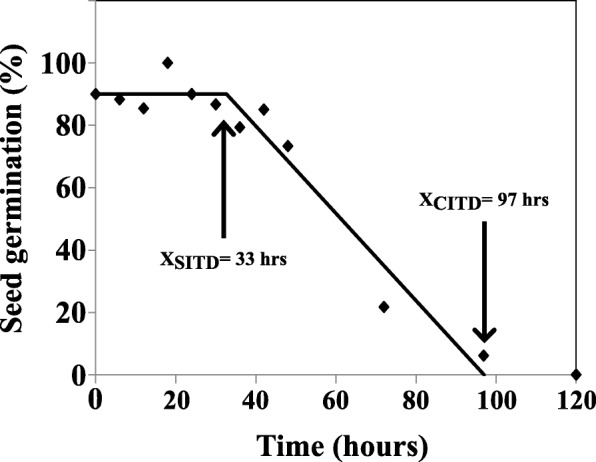
Required time to induce dormancy in tomato seeds (*Solanum lycopersicum* cv. Moneymaker)

Since we observed that high temperature induced thermo-dormancy in MM seeds, we investigated in more detail how long MM seeds required to be at high temperature to induce dormancy. During the first 33 h (~ 1.5 days) seed germination was not influenced by high temperature and thus they germinated to almost 90%. Seeds started to go into thermo-dormancy after 33 h at 37 °C. From this time point onwards seed germination declined sharply until 4 days at which seeds did not germinate anymore (Fig. 4).

### Gene expression analysis

**Fig. 5:**
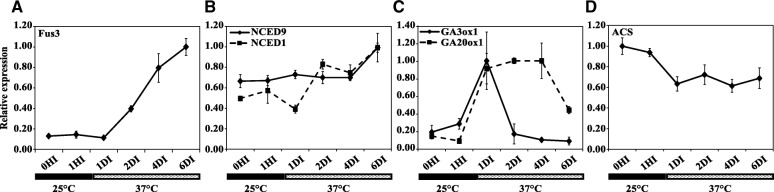
Relative expression of target genes in *Solanum lycopersicum (cv. Moneymaker)* dry seeds (0HI); Seeds imbibed at 25 °C for 1 h (1HI) and imbibed at 37 °C for 1 (1DI), 2 (2DI), 4 (4DI) and 6 (6DI) days for *fus3* (**a**), *NCED1* and *NCED9* (**b**), *GA3ox1* and *GA20ox1* (**c**) and *ACS1* (**d**)

Several genes have been implied in the regulation of thermo-inhibition and thermo-dormancy in species such as lettuce and Arabidopsis [6, 9, 30]. Since this type of dormancy has not been previously reported in tomato, we selected tomato homologs with the highest identity to Arabidopsis and lettuce genes for ethylene, ABA and GA biosynthesis pathway such as *ACS1*, *NCED9*, *NCED1*, *ABA1*, *GA3ox1*, *GA20ox1* and *GA20ox3*. Additionally, the expression of *FUSCA3* was also analysed due to its direct regulatory effect on the expression of several ABA and GA biosynthesis genes. To test whether *FUS3* is possibly associated with a role in the induction of thermo-dormancy in tomato seeds, expression levels of *FUS3* were measured during imbibition at 25 and 37 °C. *FUS3* transcript abundance was very low in dry seeds and in 1 h and 1 day imbibed seeds at 25 °C and 37 °C (Fig. 5a). However, transcript abundance was significantly increased in 4DI and 6DI seeds which already expressed thermo-dormancy (Fig. 5a). Additionally, we measured expression of some genes related to ABA and GA biosynthesis. Although *NCED5* was not expressed in the selected stages, transcript levels of *NCED9* and *NCED1* increased in seeds exposed to 37 °C (Fig. 5b). In the case of *NCED9*, expression remained fairly constant until 4 days at 37 °C (4DI) but displayed increased expression in 6DI seeds. *NCED1* transcript levels started to increase from 2 days of high temperature imbibition onwards with the highest level in 6DI seeds (Fig. 5b).

Abundance of both *GA3ox1* and *GA20ox1* transcripts was very low in dry and 1 h imbibed seeds at 25 °C but their expression levels peaked at 1 and 2 days of imbibition at 37 °C, respectively and decreased thereafter (Fig. 5c).

We measured the expression level of *ACS* which is one of the genes in the ethylene biosynthesis pathway. *ACS* transcript abundance was decreased upon imbibition at high temperature, but expression did not change further at longer durations of the high temperature treatment (Fig. 5d).

### A genetic basis of thermo-inhibition and thermo-dormancy in tomato

Similar to MM seeds those of PI germinated also around 90% at 25 °C in both light and dark conditions (Fig. 1a, b). However, under high temperature (37 °C) conditions the two genotypes showed an inverse germination behaviour. As described before, MM seeds neither germinated at light nor at dark conditions at 37 °C (Fig. 1a, b). At the same temperature light played a very critical role for germination of PI seeds. In the presence of light PI seeds germinated almost 0%, while without light their germination increased to around 90% at 37 °C (Fig. 1a, b). It is worth noting that despite the high germination percentage of the PI seeds at high temperature, they did not grow into normal healthy seedlings (Additional file 1: Fig. S2). Therefore the PI seeds showed a behaviour which could be called thermo-insensitive germination. Due to the differences observed for thermo-dormancy induction in MM versus PI, we were interested in the differences between these genotypes that cause this contrasting phenotype. To study these, we used a RIL population of 100 lines derived from a cross between MM and PI to investigate the genetic basis of thermo-dormancy in tomato seeds.

**Fig. 6:**
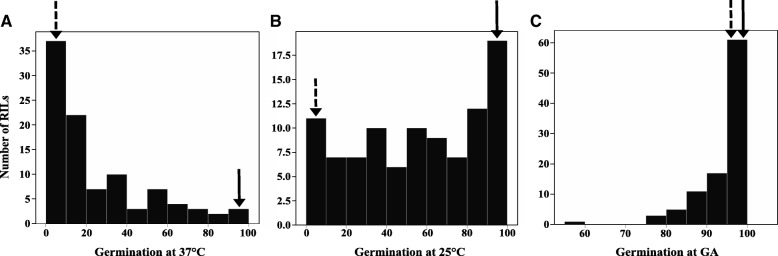
Frequency distribution of non-normalized data of the cumulative germination percentage of *Solanum lycopersicum* and *Solanum pimpinellifolium* RILs. Germination percentage of all lines was assessed in the dark: **a**, at high temperature (37 °C); **b**, at 25 °C (of the seeds that did not germinated at 37 °C); **c**, at 25 °C after using GA and stratification for remaining non-germinated seeds. The average germination percentage of parental line is indicated by a solid arrow (PI) and dashed arrow (MM)

The frequency distribution of germination percentage revealed that although germination values of the RILs varied between the two parental lines, at 37 °C and 25 °C many of the RILs either germinated to a very low percentage or did not germinate at all similar to the MM parental line (Fig. 6a, b, Additional file 1: Table S2). Exposing these non-germinated seeds to GA and stratification at 4 °C for three days resulted in almost 100% germination at 25 °C (Fig. 6c, Additional file 1: Table S2). Taken together these results illustrate that most of the progenies did not inherit the germination ability at high temperature from the PI parental line. Apparently, these lines do not possess the loci which make PI thermo-insensitive and, thus, they display thermo-dormancy. In our study we considered seeds as thermo-tolerant, thermo-inhibited and thermo-dormant when they germinated at 37 °C, at 25 °C but not at 37 °C and at 25 °C after GA and stratification treatment, respectively.

### Identification of QTLs for thermo-inhibition and thermo-dormancy in the tomato RIL population

**Fig. 7:**
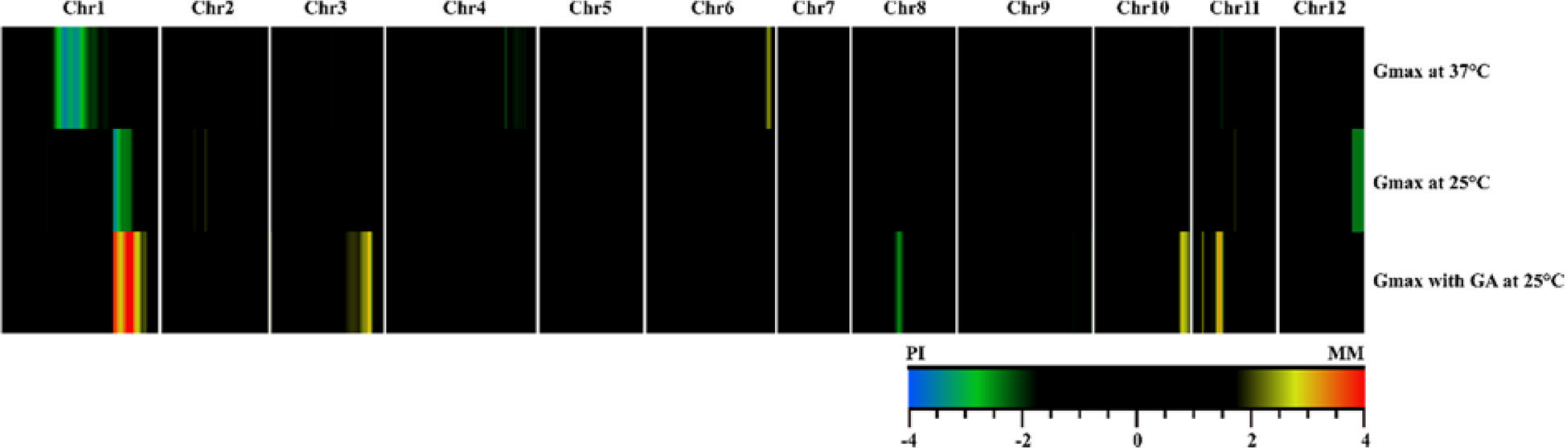
Genomic location of QTLs detected for thermo-tolerance, thermo-inhibition and thermo-dormancy of a *Solanum lycopersicum* and *Solanum pimpinellifolium* RIL population. The 12 chromosomes of tomato are separated by white lines. Centimorgan positions increase from left to right. 37 °C, 25 °C and GA represent germination at 37 °C (thermo-tolerance), 25 °C (thermo-inhibition) and 25 °C after GA and stratification treatments (thermo-dormancy), respectively. Colours across the chromosomes display significant QTLs (*P* = 0.05) based on the LOD colour scheme in which green and blue represent a larger effect of *S. pimpinellifolium* (PI) alleles on the traits and yellow and red of the *S. lycopersicum* (MM) alleles

In order to identify the loci regulating the existing diversity of seed thermo-inhibition and thermo-dormancy in MM and PI, we performed a QTL analysis using the RIL seed germination percentages at 37 °C, the following incubation at 25 °C, or further incubation at 25 °C with a pre-treatment of GA and stratification. The position, related marker, LOD score and other characteristics of the identified QTLs for thermo-inhibition and thermo-dormancy are listed in Table 1. The heat map of LOD profiles visualizes QTLs as hot spots across the 12 chromosomes of tomato (Fig. 7). In total 9 putative QTLs were detected which were predominantly related to thermo-dormancy. For this trait we found specific QTLs on chromosomes 3, 8, 10 and 11. Furthermore, a robust QTL on chromosome 1 co-located with the QTL detected for thermo-inhibition. We found one QTL regulating the thermo-tolerance trait on the same chromosome but at a different location (Table 1, Fig. 7).

**Table 1:** Characteristics of all QTLs associated with thermo-tolerance, thermo-inhibition and thermo-dormancy in a *Solanum lycopersicum* x *Solanum pimpinellifolium.* RIL population

Trait	Chromosome	Marker^1^	LOD^2^	Supporting interval (CM)	Explained variance^3^ (%)	Additive^4^
Th-T*	1	67,512,259-1	3.53	53.737–89.311	13.6	−0.104
	6	43,761,285–6	2.3	94.955–100.821	8.7	0.78
Th-I**	1	83,852,566–1	3.66	119.883–134.3	14.1	−0.094
	12	62,576,889–12	2.27	74.454–77.989	8.5	−0.074
Th-D***	1	86,171,125–1	5.45	119.883–139.883	16.3	−0.122
	3	58,231,771–3	3.24	103.485–111.308	9.2	0.092
	8	50,811,756–8	2.41	48.872–57.589	6.7	−0.081
	10	63,534,969–10	2.96	98.017–107.47	8.3	0.088
	11	47,411,518–11	3.29	20.24–25.426	9.3	0.094

*Thermo-tolerance, **Thermo-inhibition, ***Thermo-dormancy

^1^Physical position of closest marker to the QTL peak

^2^LOD score illustrating significant threshold (*P* = 0.05) calculated by a permutation test

^3^Percentage of variation explained by each QTL

^4^Positive and negative effect respectively representing the contribution of alleles of *Solanum lycopersicum* and *Solanum pimpinellifolium* to increase the traits at that specific locus

## Discussion

There are many reports on adverse effects of high temperature on seed germination of several crops such as sunflower [10], carrot [8] and lettuce [7]. Tomato, however, as one of the most important crops worldwide, has not been investigated for this adverse effect so far. Here we report on the molecular basis and mechanisms regulating secondary dormancy induced by high temperature in tomato seeds.

MM seeds displayed thermo-induced secondary dormancy regardless of light conditions (Fig. 1a, b). In addition, we found that induction of secondary dormancy in MM seeds is highly dependent on the duration of exposure to high temperature. Our results show that short term (≤33 h) exposure of MM seeds to high temperature is not sufficient for induction of secondary dormancy. Induction starts after 33 h, resulting in decreased seed germination percentages, reaching 0% after ~ 100 h.

Generally, thermo-dormancy prevents seeds from germination which needs to be relieved to allow germination. Light is one of the factors which can pre-dominantly affect thermo-dormancy. There are several reports on how light can alleviate secondary dormancy induced by different factors [6, 43, 44]. We showed that after induction of secondary dormancy in MM seeds by high temperature, light plays a vital role in breaking it. Germination of thermo-dormant seeds was promoted upon encountering an optimal temperature (25 °C) and permissive light conditions (Fig. 2). Interestingly, light is showing a dual function depending on the genetic background: i) induction of secondary dormancy in PI seeds at high temperature (Fig. 1a) and ii) breaking of secondary dormancy of MM thermo-dormant seeds at optimal temperatures (Fig. 2). Apparently light, in the presence of high temperature activates different mechanisms in MM and PI. We can speculate that light might act as an additional stress factor in PI resulting in the secondary dormancy. Contrarily light in a different genetic background (i.e. MM) works as a dormancy terminating factor at high temperature condition.

It has previously been reported that endosperm weakening is one of the basic inhibiting factors for tomato seed germination [15]. Furthermore, in some crops like lettuce and tomato it has been suggested that secondary dormancy might be related to the seed coat and endosperm tissues which could be circumvented by removing either or both [45, 46]. However, we show that the endosperm does not play a role in secondary dormancy induction in MM tomato seeds, since thermo-induced secondary dormancy was not released upon removal of the endosperm and seed coat (Fig. 3). This indicates that thermo-induced secondary dormancy in tomato is a physiological process which supposedly is regulated by physiological blocks in the embryo. Despite results from previous studies on secondary dormancy in tomato pointing towards the physical inhibitory role of the seed coat (the strength of which is regulated by the physiology of the seed) in this phenomenon [15, 46], our results suggest that the thermo-dormancy induced in tomato seeds is not related to the inhibition of endosperm weakening but to embryo dormancy.

Our results suggest that induction of secondary dormancy by high temperature in tomato is significantly affected by the genetic background as MM seeds are more sensitive to high temperature than those of PI (Fig. 1). However, exposure of PI seeds to the combination of high temperature and light also resulted in the induction of secondary dormancy (Fig. 1).

Expression analysis of genes previously described to be involved in thermo-induced secondary dormancy in other species (i.e. Arabidopsis and lettuce) provided useful information on the molecular background of thermo-induced secondary dormancy in tomato. Among all investigated genes *FUS3*, as one of the master regulators of both GA and ABA biosynthetic pathways, showed a very distinct expression profile (Fig. 5a). This is in accordance with Chiu et al., [30] who demonstrated a similar regulatory role of *FUS3* in thermo-induced secondary dormancy in Arabidopsis. It has been reported that downstream genes of ABA and GA biosynthesis and catabolism (*NCED*s and *GA3ox*s and *GA20ox*s) are being regulated in accordance with the gene expression profile of *FUS3* [31, 32]. Interestingly, in the present study *GA3ox1* and *GA20ox1*, involved in biosynthesis of GA, were downregulated upon upregulation of *FUS3*. On the other hand, ABA biosynthesis pathway genes were upregulated with upregulation of *FUS3*, albeit to a lesser extent. However, in case of *NCED9*, one of the downstream ABA biosynthetic pathway genes, the expression pattern is not exactly matching to what was observed for the upstream master regulator, *FUS3*. This could be due to delayed expression of downstream genes, regulated by this transcription factor. We propose GA and ABA biosynthetic pathway genes and their master regulator(s) as putative targets to obtain further insights in this process.

Our findings regarding the effect of several GA and ABA metabolic pathway genes on high temperature-induced dormancy in tomato seeds was in accordance with previous expression analysis of similar genes in Arabidopsis [9, 30, 47, 48].

In order to further identify genes involved in the regulation of dormancy induction in tomato seeds, we have used a QTL analysis approach with a RIL population derived from a cross between the two tomato accessions *S. lycopersicum* cv. Moneymaker and *S. pimpinellifolium* accession CGN14498. Some interesting QTLs were identified (Fig. 7). The same population has previously been screened for germination QTLs under control conditions [49], but except for the QTL on chromosome 6 there was no overlap with the QTLs found in this study. It is intriguing that none of the genes (*FUS3*, *NCED9*, *NCED1*, *GA3ox1* and *GA20ox1*) commonly considered to be involved in induction of thermo-dormancy in other species [1] (Fig. 5) were co-locating with these identified QTLs. Similar holds true for the tomato homolog of ETHYLENE RESPONSE FACTOR1 (ERF1) which has been found to have a role in thermo-dormancy in lettuce [50]. Evidently, other mechanisms and/or genes may be involved in the regulation of thermo-dormancy in tomato seeds. Furthermore, the high number of identified QTLs suggests a complex multi-genic trait. QTLs identified in this research can pave further routes towards detailed investigations of the mechanism of action of thermo-dormancy in tomato seeds. Combination of fine mapping of detected QTLs combined with RNA-sequencing data will result in better understanding of this process in tomato seeds.

## Conclusion

Global warming is an undeniable phenomenon which affects slowly, but continuously, agricultural commodities. Hence understanding the underlying mechanisms in plants by which plants/seeds tolerate suboptimal temperatures is of a great importance. In this study we showed that thermo-induced secondary dormancy in tomato seeds is genotype-dependent. We observed that secondary dormancy was only induced in MM seeds encountering high temperature (37 °C) and not in PI. The induced dormancy in MM seeds was not related to the physical inhibitors such as seed coat and endosperm. A candidate gene approach has been used to check whether the molecular pathways involved in thermo-induced dormancy in tomato are similar to the ones in other species such as lettuce and Arabidopsis (ABA and GA). Upregulation of ABA biosynthesis pathway genes (*NCED1* and *NCED9*) and on the other hand downregulation of two of the GA-biosynthesis genes (*GA3ox1* and *GA20ox1*) in tomato thermo-dormant seeds at elevated temperature implies similar mechanisms as the reported ones of lettuce and Arabidopsis involved in thermo-dormancy in tomato seeds. Besides, QTL analysis showed genomic regions involved in thermo-dormancy regulation. Intriguingly, the mentioned regulatory molecular elements in thermo-dormancy (*NCED*s and *GAox*s) were not co-located with our detected QTLs. This finding points towards additional mechanisms involved in tomato seeds thermo-dormancy regulation. Identification of genes causal for these QTLs and their functional characterization will pave the route towards identification and characterization of those mechanisms.

## Methods

### Plant material

The RIL population was obtained from a cross between two parental lines: *S. lycopersicum* cv. Moneymaker and *S. pimpinellifolium* accession G1.1554 [42]. This population was provided and produced by Adriaan W. van Heusden of Wageningen UR Plant Breeding, Wageningen, The Netherlands. *S. lycopersicum* cv. Moneymaker and *S. pimpinellifolium* accession G1.1554 were obtained from the Centre Genetic Resources: the Dutch genebank for plant genetic resources for food and agriculture under a mandate of the Netherlands government (reference CGN14330 and CGN14498, respectively). A total of 727 single nucleotide polymorphism (SNP) markers was used for genotyping the population in F_7_ and seeds of F_8_ plants were used for the phenotyping. The RIL population, together with the parental lines, were grown under standard conditions in a greenhouse at 25 °C and 15 °C during day and night and 16 and 8 h of light and dark, respectively. The fully ripened fruits were harvested and the seeds were extracted using 1% hydrochloric acid (HCl) to remove all sticky parts of the seed’s pulp. Afterwards, the seeds were soaked in a trisodium phosphate (Na_3_PO_4_.12H_2_O) solution to disinfect the seed batches. Finally, the seeds, which had been dried at room temperature for 3 days, were stored in paper bags under cool and dry conditions (13 °C and 30% RH) [49].

### Seed germination assay

In order to study how temperature and light regulate seed germination of the two parental lines (MM and PI), three replications of around 50 seeds were sown on germination trays (21 × 15 cm DBP Plastics, http://www.dbp.be). Each tray contained two layers of blue germination paper (5.6′ × 8′ Blue Blotter Paper; Anchor Paper Company, http://www.seedpaper.com) and 50 ml demineralized water. Trays were piled with one empty tray consisting of one germination paper and 50 ml of water at the bottom and top of the pile to prevent unequal evaporation. The piles were transferred to either optimal (25 °C) or high temperature (37 °C) in both presence and absence of light. Germination was scored manually twice per day for one week at optimal as well as high temperature.

### Germination of tomato embryos

MM seeds were imbibed at 25 °C for 3–4 h and subsequently seed coat and endosperm layers were removed using forceps and a sharp blade. The extracted embryos were immediately placed on new germination trays with 2 blue germination papers and 50 ml water. The trays were transferred to optimal (25 °C) and high temperature (37 °C) in dark. The growth of the radicles was evaluated manually once a day for 6 days.

### Time required to induce dormancy

Seeds of MM were subjected to 37 °C for 6, 12, 18, 24, 30, 36, 42, 72, 96, 120 and 144 h. Subsequently, they were transferred to 25 °C for 14 days. Afterwards, the normal germinated seeds were counted manually for each time point. A segmented model was fitted to seed germination (percentage) versus time (hours) using the NLIN procedure of the SAS software [51]:

$$ \mathrm{y}=\mathrm{a}+\mathrm{b}\ast \mathrm{x}\ \mathrm{for}\ \mathrm{x}>{\mathrm{x}}_0 $$

$$ \mathrm{y}=\mathrm{a}+\mathrm{b}\ast {\mathrm{x}}_0\ \mathrm{for}\ \mathrm{x}<{\mathrm{x}}_0 $$

where y is germination percentage, x is days at 37 °C, b is model slope line, x_0_ is the start time of dormancy induction.

### Effect of light on thermo-dormancy alleviation

MM seeds were imbibed at 37 °C for 4 and 6 days and then transferred to 25 °C under light and dark conditions. Germinated seeds were counted every day for 18 days. Since the seeds did not germinate in the dark, they were transferred to light after 18 days of dark imbibition and seed germination was scored every day for 10 days. For each condition a logistic model was fitted to the cumulative seed germination (percentage) versus time (days) with the NLIN procedure using SAS software [52].

$$ \mathrm{y}\left(\%\right)={\mathrm{G}}_{\mathrm{max}}/\left(1+{\left(\mathrm{x}/{\mathrm{D}}_{50}\right)}^{\wedge }{\mathrm{G}}_{\mathrm{rate}}\right) $$

where y is the total germination (%) at time x, G_max_ is the maximum germination (%), D_50_ is the time to 50% of the maximum germination and G_rate_ indicates the slope of the curve at D_50_.

### Germination assays for QTL analysis

The seeds of the RIL population were sown in 4 replications as described above for the germination assay of the parental lines. Following stratification at 4 °C, the trays were transferred to 37 °C and kept there for five days. After this incubation the healthy germinated seeds were counted and considered thermo-insensitive. Thereafter, the non-germinated seeds were incubated at 25 °C for one week after which the geminated seeds were manually scored and considered thermo-inhibited. Remaining non-germinated seeds were transferred to new trays containing two layers of blue germination paper and 10 μM GA which were incubated at 4 °C for 3 days. After this stratification the trays were incubated at 25 °C and eventually the final germinated seeds were scored again after 5 and 7 days and considered thermo-dormant. QTL detection for the traits under study was carried out with simple interval mapping (SIM) using mapping software MapQTL® 6.0 based on the linkage map of the RIL population (Additional file 2) containing 727 SNP markers [53]. Thousand permutation tests of our data were implemented in MapQTL^®^ 6.0 and resulted in a 95% LOD threshold of 2.0. Therefore, we adjusted the LOD threshold to 2.0 to determine putative QTLs related to thermo-inhibition and thermo-dormancy [54].

### RNA extraction and cDNA synthesis

Total RNA was extracted from 30 seeds of each sample. Dry seeds, imbibed seeds at 25 °C for 1 h and imbibed seeds at 37 °C for 1, 2, 4 and 6 days were used for RNA extraction. The seeds were frozen in liquid nitrogen and ground by a dismembrator (Mikro-dismembrator U; B. Braun Biotech International, Melsungen, Germany), with the help of 1 1/8 in. RNAse free metal bullet at 2000 rpm for 1 min. Then, 1.5 ml of buffer A containing 681 μl of a mix of TLE grinding buffer and β-mercaptoethanol, 681 μl phenol and 138 μl chloroform, was added to each sample and mixed immediately. The TLE grinding buffer consisted of Tris (0.18 M) (Trizmabase Fulka 3362), LiCl (0.09 M) (Sigma L0505), EDTA (4.5 mM) (Sigma E-5134), SDS (1%) (natriumlaurylsufaat Sigma L3771). The mixture of homogenized sample and buffer A was centrifuged for 10 min at maximum speed (14,000 rpm) and the supernatant was collected subsequently and placed in a new 2 ml microfuge tube. One ml of 1:1 phenol:chloroform was added, followed by vortexing and centrifugation for 2 min at 14000 rpm. Afterwards, 1 ml of chloroform was added to the collected supernatant, which was then placed in a new microfuge tube, and mixed and centrifuged at 14000 rpm for 2 min. The new supernatant was collected again and transferred to a new tube and thereafter 100 μl of 10 M LiCl was added, mixed well, and stored at 4 °C on ice overnight. The following day, thawed samples were centrifuged at 4 °C for 30 min at 14000 rpm, followed by pipetting off the supernatant, adding 250 μl of 70% cold ethanol to the remaining pellet and shaking. After 5 min centrifugation at 14000 rpm at 4 °C, the supernatant was removed and the remaining pellet was air dried for 10 to 15 min in a fume hood. The dried pellet was dissolved in 30 μl RNAse free water and stored at − 80 °C. RNA was quantified spectrophotometrically using a QIAxpert device (www.qiagen.com/nl/resources/knowledge-area/reproducibility-throughautomation/qiaxpert/). RNA integrity was further qualified by checking the ribosomal RNA bands on a 1% agarose gel. Samples with sharp and clear bands without obvious degradation were selected for subsequent steps. Five μg of RNA from each sample was treated with DNAse by adding 10 μl of DNAse enzyme (Promega) and DNAse buffer, filled to 100 μl with RNAse free water and incubated at 37 °C for 30 min. Subsequently 100 μl of phenol:chlorophorm (1:1) was added and the solution was transferred to phase lock tubes and centrifuged for 5 min at 14000 rpm. After centrifugation the supernatant (~ 90 μl) was collected, placed in a new tube and 9 μl of 3 M NaAc and 250 μl of 100% ice-cold ethanol were added and kept at − 20 °C for 2 h. After 2 h the tubes were centrifuged for 30 min at 4 °C and 14,000 rpm and, subsequently, the supernatant was removed and the remaining pellet was washed with 250 μl of 70% cold ethanol and centrifuged for 5 min at 4 °C. In the final step, after removing the supernatant, the pellet was air dried for 10–15 min and dissolved in 20 μl of RNAse free water. cDNA was synthesized from 500 ng of total RNA according to the manufacturer’s protocol (iScriptTM cDNA synthesis kit, Bio-Rad) and diluted 20 times with miliQ water and stored at − 20 °C.

### Reference and target gene selection, primer design and RT-qPCR analysis

*TIP41-like* (SGN-U584254) and *PP2Ac1* (SGN-U567355) were used as reference genes [55]. CLCbio software (CLCbio, Aarhus, Denmark) was used to design the primers with melting temperature of 58–62 °C, a length of 18–22 bp and a template length of 80–200 bp. The efficiency of the primers was evaluated with a two-fold series dilution of a pooled cDNA of all samples. Gene names and their homologs and annotation and primer sequences are described in Additional file 1: Table S1. The RT-qPCR was performed using 2.5 μl of cDNA, 0.5 μl of primer mix (forward and reverse), 5 μl of iQ SYBR Green Supermix (Bio-Rad) and 2 μl of miliQ water according to the manufacturer’s instructions (CFX, Bio-Rad). The RT-qPCR protocol used for the analysis was 95 °C for 3 min, continued with 40 cycles of 95 °C for 15 s and 60 °C for 30 s and melting curves were recorded [56].

## Additional files

Additional file 1: **Table S1.** Description of target genes and primers used for RT-qPCR. **Table S2.** Average seed germination of RILs and the parental accessions *Solanum lycopersicum* (cv. Moneymaker) and *Solanum pimpinellifolium*. **Figure S1.** Germination of tomato embryos (*Solanum lycopersicum* cv. Moneymaker) at normal (25 °C) and high temperature (37 °C) at first, third and sixth day after sowing. **Figure S2.** Germination of *Solanum pimpinellifolium* seeds at 37 °C at first, second, third, fourth and fifth day after sowing. (DOCX 244 kb)

Additional file 2: The linkage map of the RIL population. (XLSX 31 kb)

## Abbreviations

ABA: Abscisic acid
ACC: 1-aminocyclopropane-1-carboxylic acid
*ACS*: 1-aminocyclopropane-1-carboxylic acid synthase
DI: Days after imbibition
FUS3: FUSCA3
GA: Gibberellic acid
*GA20ox1*: GIBBERELLIN 20-OXIDASE 1
*GA3ox1*: GIBBERELLIN 3-OXIDASE 1
LOD: Logarithm (base 10) of odds
MM: *Solanum lycopersicum* (cv. Moneymaker)
*NCED1*: 9-cis-epoxycarotenoid dioxygenase 1
*NCED9*: 9-cis-epoxycarotenoid dioxygenase 9
PI: *Solanum pimpinellifolium*
QTL: Quantitative trait loci
RIL: Recombinant inbred line
SNP: Single nucleotide polymorphism
