# Supplementary material for: Characterization of and genetic variation for tomato seed thermo-inhibition and thermo-dormancy
Source: BMC Plant Biol. 2018 Oct 11;18:229. doi: 10.1186/s12870-018-1455-6 (PMC6182833; doi:10.1186/s12870-018-1455-6)
Supplement: Supplementary file 1 — Table S1. Description of target genes and primers used for RT-qPCR. Table S2. Average seed germination of RILs and the parental accessions Solanum lycopersicum (cv. Moneymaker) and Solanum pimpinellifolium. Figure S1. Germination of tomato embryos (Solanum lycopersicum cv. Moneymaker) at normal (25 °C) and high temperature (37 °C) at first, third and sixth day after sowing. Figure S2. Germination of Solanum pimpinellifolium seeds at 37 °C at first, second, third, fourth and fifth day after sowing. (DOCX 244 kb) [file 12870_2018_1455_MOESM1_ESM.docx]

**Supplemental files**

| **Table S1.** Description of target genes and primers used for RT-qPCR. | | | | |
| --- | --- | --- | --- | --- |
| **Gene** | **Homologs** | **Froward** | **Reverse** | **Amplicon length** |
| ***NCED1*** | Solyc01g087250.2 | GGCAGATTTGGTTCGGAGG | GCTGACTTTCCGGTGTTCT | 112 |
| ***NCED5*** | Solyc08g016720.1 | AGTGAGCTATTCTTGCCGT | CTACAAGCCCGAAAACTCC | 157 |
| ***NCED9*** | Solyc08g016720.1 | GGGAAGAACCAGAAACAGATGA | AGGGTGAAAGTAGTTGTCGT | 163 |
| ***GA3ox1*** | Solyc06g066820.2 | AGATGATGTAAAATGGGCCGT | AGAATCCGTATGTGCTGCAA | 130 |
| ***GA20ox1*** | Solyc07g056670.2 | CTTCTCCTCTCCCCTCTCT | CCGCAGCCCGAATGTTTT | 191 |
| ***ACS*** | Solyc12g056180.2 | AGGAAAGCTTATTGGGGTTGT | GGCTTCATGTTCTCGCATT | 99 |
| ***FUS3*** | Solyc02g094460.2 | GCTGCAGATGATGGGAATGGA | ACAAAGTCGAAGGCAGGC | 143 |

| **Table S2.** Average seed germination of RILs and the parental accessions *Solanum lycopersicum* (cv. Moneymaker) and *Solanum pimpinellifolium*. | | | |
| --- | --- | --- | --- |
| **Trait** | **MM** | **PI** | **RIL** |
| **G_max_ at 37°C** | 5 | 98 | 25 |
| **G_max_ at 25°C** | 40 | 2 | 26 |
| **G_max_ with GA at 25°C** | 55 | 0 | 44 |
| **G_max_ at 37°C**, Germination at 37°C; **G_max_ at 25°C**, Subsequent germination at 25°C; **G_max_ with GA at 25°C**, Germination of previously non-germinated seeds after treatment with GA and stratification at 25°C; **MM**, *Solanum lycopersicum* (cv. Moneymaker); **PI**, *Solanum pimpinellifolium*, **RIL**, Recombinant Inbred Lines. | | | |

| 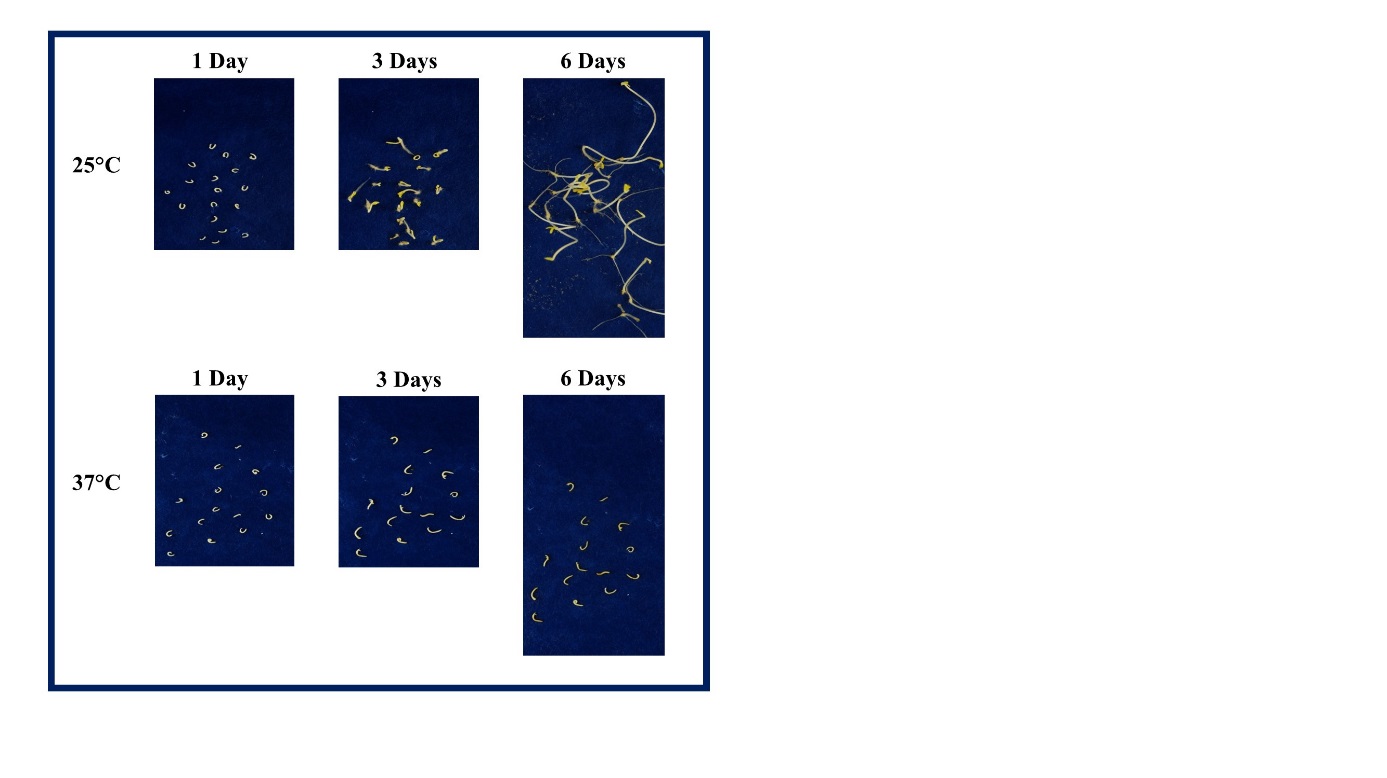 |
| --- |
| **Figure S1.** Germination of tomato embryos (*Solanum lycopersicum* cv. Moneymaker) at normal (25°C) and high temperature (37°C) at first, third and sixth day after sowing. |

| 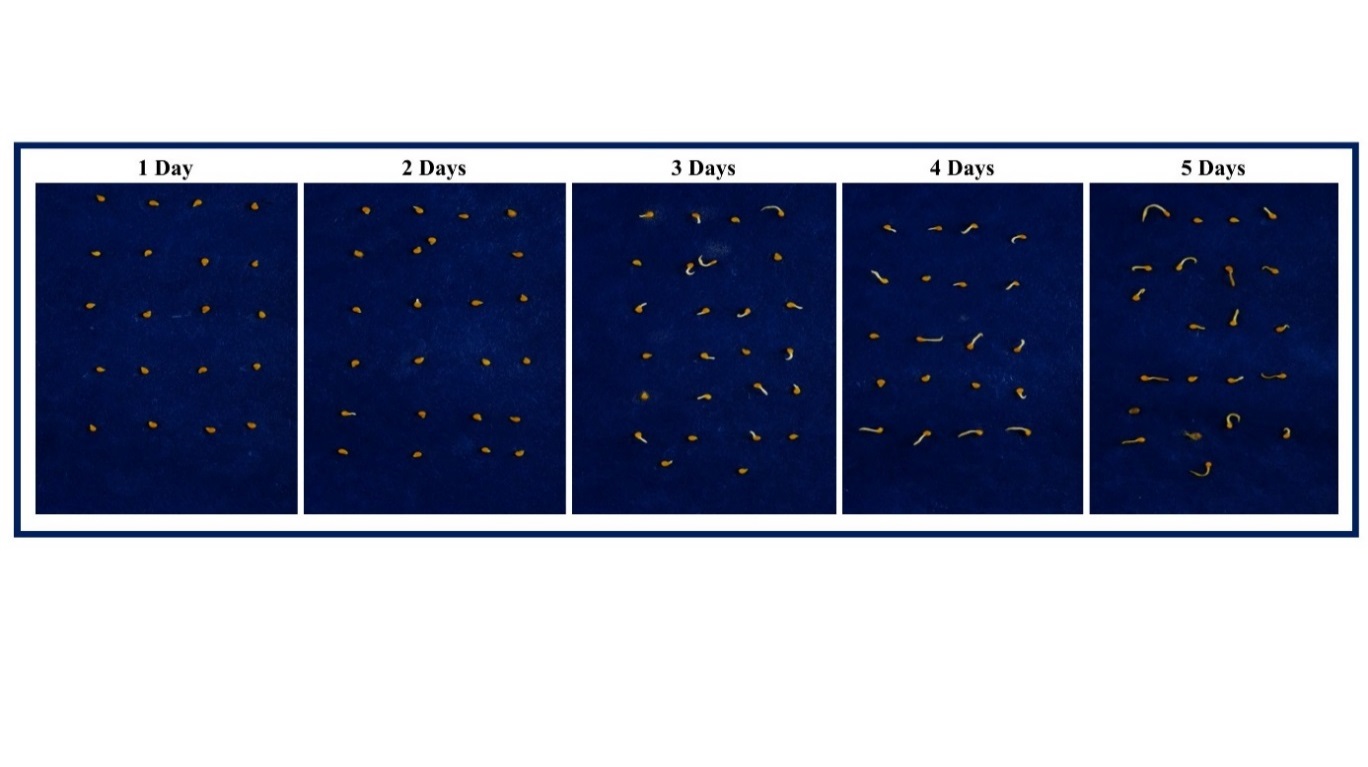 |
| --- |
| **Figure S2.** Germination of *Solanum pimpinellifolium* seeds at 37°C at first, second, third, fourth and fifth day after sowing. |
